# Is the Long-Term Use of Benzodiazepines Associated With Worse Cognition Performance in Highly Educated Older Adults?

**DOI:** 10.3389/fpsyt.2020.595623

**Published:** 2020-10-26

**Authors:** Linzi Liu, Peiying Jian, Yifang Zhou, Jian Zhou, Linna Jia, Minghui Tang, Rongwei Zhang, Yanqing Tang

**Affiliations:** ^1^Department of Psychiatry, The First Affiliated Hospital, China Medical University, Shenyang, China; ^2^Department of Psychology, Queen's University, Kingston, ON, Canada; ^3^Department of Geriatrics, The First Affiliated Hospital, China Medical University, Shenyang, China; ^4^The Clinical College of Precision Medicine, Liaoning He's Medical College, Shenyang, China

**Keywords:** cognition, benzodiazepines, elderly, education, sedative-hypnotics

## Abstract

**Background:** Benzodiazepines (BZD) are common medications for sedative, hypnotic, and anxiolytic that are especially prevalent in older adults. Previous studies have shown that BZD use could impair users' cognition, significantly affecting their quality of life. Past research has shown that higher education might play a protective role in the process of cognitive decline. Very few studies had examined the cognitive effects of BZD on highly educated older adults. The study aimed to explore how cognitive functions would be affected by benzodiazepines among highly educated older adults.

**Method:** 140 older adults with an average education period of 14.8 years were included in this study. The subjects were divided into three separate groups, the long-term BZD users (≥180 days), short-term BZD users (<180 days), and non-users. Demographics and cognitive assessments for the three groups were analyzed using the analysis of variance (ANOVA), the chi-squared test, and the analysis of covariance (ANCOVA). To examine the association between BZD use and cognition a multiple linear aggression approach was used.

**Result:** All three groups were significantly different from each other when looking at executive functioning in the Trail Making Test B (TMT-B). Compared to the control group, short-term BZD users showed significant defects in TMT-B time (*p* = 0.002) and TMT-B errors (*p* < 0.001); long-term BZD users showed significant defect on TMT-B time (*p* = 0.041). Compared to short-term BZD users, long-term BZD users showed significant merit on TMT-B errors (*p* = 0.001). No significant differences were found in other cognitive tasks that reflected general cognition, verbal memory, language fluency, and visual memory. After adjusting for demographic, increased BZD use over time was positively associated with scores for the revised Brief Visuospatial Memory Test (*r* = 0.377, *p* = 0.012).

**Conclusion:** BZD use may be significantly associated with worse executive functioning in highly educated older adults. However, there is no association between the duration of BZD use and increased cognitive deficits in highly educated older adults. This study identified future experimental directions including potential longitudinal studies, within-subject studies comparing mood disorder patients' cognitive performance before and after onset of BZD use, and between-subject studies that directly compare BZD's effect on subjects with the same baseline of cognitive functioning.

## Introduction

Benzodiazepines (BZD) are a class of psychoactive drugs that can enhance the binding of the neurotransmitter gamma-aminobutyric acid (GABA) on the GABA-A receptor, resulting in sedative, hypnotic, and anxiolytic effects (1). BZDs have been used for the treatment of a wide range of clinical conditions such as anxiety, insomnia, and alcohol dependence. Treatment using BZD has increased over time, with a significant number of older adults being prescribed BZD as their main treatment option (2). Increased prescribing of BZD is due to the high prevalence of pain, anxiety, and insomnia in this age group (2, 3). Research has suggested that BZD use was positively correlated with age (4, 5), with approximately 9–12% of old adults in the United States using BZD (6). Developed countries have reported a higher prevalence of BZD use with roughly 7–43% of older adults using BZD (7). Globally, BZD use is approximately 10–42% older adults (8–10).

In recent years, several studies have shown that BZD use may impact the cognitive functioning of older adults, but studies varied in the cognitive domains observed and the participant's demographics. A cohort study by Paterniti et al. (11) using 1,389 old adults with an average education level of 10 years examined the relationship between BZD use and cognitive functioning. The results showed that long-term BZD users were at a higher risk for reduced performance in general cognition tasks for executive functioning than non-BZD users (11). A study by Bierman et al. (12) also suggested that general cognition, executive functioning, and memory in older adults was negatively affected by BZD use. Participants utilized in this study had an average age of 69 years with varied education levels (60.3% participants with low-level education, 27.3% participants with medium-level education, 14.4% participants with high-level education) (12). However, research has also suggested that there are no significant changes in cognitive functioning following BZD use in older adults. Research conducted by Hanlon et al. found that performance on cognition tasks was not associated with BZD use in individuals over 65 with different education levels (33.7% participants <8 year, 36.7% participants 8–12 year, 29.6% participants >12 year) (13). Zhang et al. found no evidence to suggest a relationship between BZD use and decline in general cognition in older adults with an average education level of 15 years (14). Consistently, Grossi et al. found no significant association between BZD use and dementia among older adults with 10 years of education (15). To sum up, research on the impact of BZD on the cognitive function in older adults were inconsistent with the large heterogeneity for demographics.

During the process of aging, structural, and functional neurological changes that occur renders older adults more vulnerable to risk factors such as psychotropic drugs, stress, and negative mental health states. However, higher education level is a protective factor of age-related cognitive decline. It has been widely accepted that higher education is consistently related to better cognitive performance in the older adults (16, 17). The protective effect of high education on cognition can be explained by the cognitive reserve hypothesis (18, 19), which suggests that people with higher cognitive reserves are more resilient to age-related neurological changes, through the maintenance of more intact cognitive functioning (20). Evidence suggests that a higher educational level is associated with more cognitive reserve (21). With the development of the economy, there was a worldwide expansion of higher education since the twentieth century (22), which led to an increased population of highly educated older adults. Based on the popularity of BZD prescription in the older adults, there would be more highly educated individuals taking BZD than ever. Research focusing on BZD's cognitive effects on highly educated older adults may provide not only evidence for the security of BZD, but also guidance on clinical application in medicine. Moreover, it may provide a new perspective to explore the protective effect of education on cognition, allowing researchers to further examine the cognitive reserve theory.

Unfortunately, very few studies have examined the effect of BZD on cognition, especially for specific cognitive domains, such as memory and executive function, of highly educated older adults. Moreover, the literature currently lacks information on the cognitive effects of BZD in highly educated older adults, and if the duration of BZD use plays a role in cognitive performance. The purpose of this study is to explore if the cognitive function of highly educated older adults is associated with BZD use and how the cognitive effect changes with the duration of BZD use. If higher education level has protective effects on cognitive performance, we expect to see a decrease in lower cognitive performance in the BZD use groups compared to the non-use group. As well, we expect that as BZD use duration increases, there will be no significant decline in cognitive scores as BZD.

## Method

### Subjects

Participants were recruited from inpatient, outpatient, and annual medical examination services at the First Affiliated Hospital of China Medical University, Shenyang, China from May 2019 to December 2019. This study was approved by the ethics committee in the First Affiliated Hospital of China Medical University. All individuals used in this experiment signed the informed consent.

The inclusion criteria were (1) aged 60 or older, (2) with a more-than-or-equal-to a high school education level. Participants were excluded if (1) they met the Diagnostic and Statistical Manual of Mental Disorders, Fifth Edition (DSM-V) diagnostic criteria for Schizophrenia Spectrum and other Psychotic Disorders, and Neurocognitive Disorders, (2) were in a state of anxiety (the Self-Rating Anxiety Scale, SAS score≥20) (23) or depression (Geriatric Depression Scale, GDS scores≥15) (24), (3) were taking drugs that affect cognitive performance such as cholinesterase inhibitors and memantine, (4) were in other statuses of abnormal cognition such as head trauma, and (5) were unable to complete cognitive assessments such as severe visual or hearing impairment, illiteracy, or color blindness. These exclusion criteria were set to ensure that the participants were not subject to other conditions that might confound cognitive performance. After screening, 140 individuals aged more than 60 years (females *N* = 51) were included in the study.

### Procedure

#### Screening

After signing the consent form, participants were evaluated for anxiety and depression symptoms using the SAS and GDS. BZD users are more likely to experience depression and anxiety than the non-users (25). However, depression and anxiety can significantly affect cognitive test performance, confounding the result of the study (26). To reduce the influence of depression and anxiety on the cognition results in this study, participants with moderate symptoms of anxiety or depression, reflected by scores of SAS score of ≥20 and GDS scores of ≥15, respectively, were excluded. Participants not in a state of anxiety or depression then participated in face-to-face interviews to collect demographic and clinical information including age, sex, education, height, weight, smoking status, drink status, medication information, and chronic disease history. Participants' heights and weights were collected to calculate the body mass index (BMI). The information on medication and chronic disease history was supplemented by the electronic medical record system of the First Affiliated Hospital of China Medical University and the physical examination system of the geriatrics department of the First Affiliated Hospital of China Medical University. A total of 140 participants remained after screening with the exclusion criteria. The demographic and clinical information of the included participants are presented in Table 1.

**Table 1:** Demographic information of the participants.

	**Comparison group (*n* = 47)**	**Short-term using group (*n* = 46)**	**Long-term using group (*n* = 47)**	***P-value***
Sex, male/female	30/17	31/15	28/19	0.735
Age, year, mean ± SD	71.40 ± 8.40	74.93 ± 10.78	75.06 ± 10.25	0.129
Education, year, mean ± SD	15.47 ± 2.67	14.11 ± 3.76	15.07 ± 2.92	0.103
Smoke, yes/no	5/42	5/41	7/40	0.778
Drink, yes/no	6/41	3/43	5/42	0.348
BMI, kg/m^2^, mean ± SD	24.30 ± 2.73	24.15 ± 2.60	23.00 ± 3.06	0.036^*^
Cerebrovascular disease, yes/no	17/30	20/26	25/22	0.249
Hypertension, yes/no	29/18	31/15	29/18	0.806
Coronary heart disease, yes/no	17/30	22/24	22/25	0.232
Diabetes, yes/no	19/32	12/34	18/29	0.295

BMI, Body Mass Index;

* *P < 0.05*.

#### BZD Use Groups and Controls

This study included all classes of benzodiazepines: anxiolytic (N05BA), hypnotic and sedative (N05CD), antiepileptic (N03AE), and myorelaxant (M03BX07). Hypnotic drugs derived or connected with benzodiazepines (N05CF) were also included. Based on the self-report medication history obtained in the interviews and prescription record in the electronic medical record system, we converted the accumulated BZD dosage and average daily BZD dosage of each participant into diazepam equivalents (27). Based on the BZD use behavior provided in the participants medical information, the participants were divided into three BZD use groups: long-term BZD users (*N* = 47, BZD using time ≥180 day), and short-term BZD users (*N* = 46, BZD using time <180 day), and non-users (*N* = 47, never used BZD) (28).

#### Cognitive Performance Tests

The cognitive function evaluation process consisted of six cognitive tasks reflecting general cognition and four subdomains of cognition: memory (visual memory and verbal memory), attention, executive function, and verbal fluency. Cognitive tasks were selected based on high credibility among older adults in China (29).

*The Mini-Mental State Examination test (MMSE)* (30, 31) is a widely used cognition evaluating instrument. It includes the assessment of multiple cognitive dimensions. The test was used to assess the participants' general cognitive levels and excluded dementia patients.

*The Rey Auditory Verbal Learning Test (AVLT)* (32, 33) has multiple measures of memory and was easy to apply. This test was used to evaluate the participants' functions of verbal memory.

*The Trail Making Test (TMT)* (33, 34) is a commonly used executive function detection tool and plays an auxiliary role in the identification of dementia and early stage of cognitive impairment. TMT-A assesses attention and visuomotor processing speed, while the TMT-B assesses attention and visuomotor processing speed, visual-motor ability, and working memory (35, 36).

*The Controlled Oral Word Association Test (COWAT)* (37, 38) is a widely used verbal fluency test. The purpose of the test is to evaluate the spontaneous production of words within a limited amount of time. This test is sensitive to language function.

*The Digit Span Test (DST)* (33, 39) is often used to test short-term attention function and is widely used in geriatrics research.

*The Brief Visuospatial Memory Test-Revised (BVMT-R)* (40, 41) are widely used tests of visual memory in both clinical and research settings.

### Data Analysis

IBM SPSS Statistics for Windows, Version 22 was used for all analyses. The analysis of variance (ANOVA) tests were used to compare the continuous demographic variables. Chi-square tests were used to compare the classified demographic variables and chronic conditions history of disease among the two BZD use groups and the control group. These statistical tests on the demographic information aimed to identify potential covariates among the three BZD use groups. BZD use information was determined with descriptive statistics. In order to evaluate the cognitive performance among the three BZD use groups, a comparison of cognitive task results was conducted using analysis of covariance (ANCOVA) was performed with BZD use group as a fixed effect adjusting for the demographic variables. Furthermore, to investigate whether BZD use duration can predict cognitive performance, we conducted a bivariate analysis and a multiple linear regression analysis within the long-term and short-term BZD use groups using participants' BZD use duration and cognitive tasks results. The covariates included in the model were age, gender, education, and BMI. A significance level of 0.05 was used for all tests of significance.

## Result

The descriptive demographic information is shown in Table 1 and the BZD use is shown in Table 2. A total of 140 subjects were included in this study: 51 females and 89 males. The average age of all subjects was 73.79 ± 9.94 years, and the average number of years in education was 14.88 ± 3.19 years. The three groups were matched for gender, age, education years, smoking, drink, and history of chronic diseases. There was a significant difference between groups in the BMI (*p* = 0.036). Compared to the non-users and short-term BZD users, the BMI of the long-term BZD use group is significantly lower. Among the 93 BZD users, the average accumulated dose of BZD users was 498.13 diazepam equivalent, the average daily dose of BZD users was 0.71 diazepam equivalent, and the average duration of medication was 649.69 days. Seventeen BZD users took BZD because of anxiety and the other 76 participants use it for insomnia.

**Table 2:** BZD use information of the participants.

	**Short-term BZD users (*n* = 46)**	**Long-term BZD users (*n* = 47)**
Accumulated BZD using dosage, diazepam equivalent, mean ± SD	0.50 ± 0.61	0.92 ± 1.53
Average BZD using dosage, diazepam equivalent, mean ± SD	31.27 ± 38.06	955.06 ± 1578.98
BZD using time, d, mean ± SD	65.72 ± 42.58	1221.23 ± 1556.04

**Figure 1:**
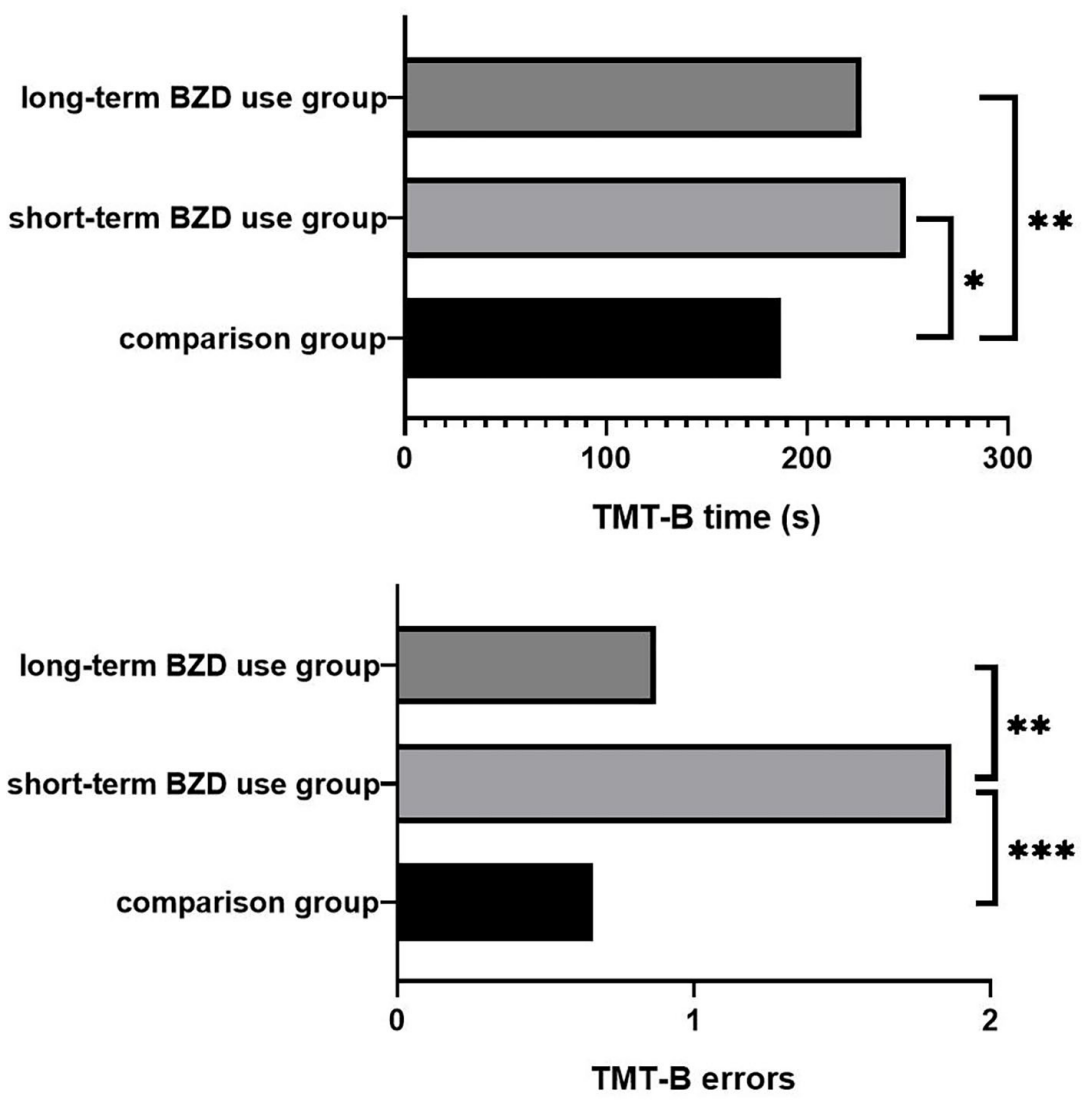
Post-comparison of TMT-B results between groups. *p < 0.05; **p < 0.005; ***p < 0.001.

The results of the ANCOVA for differences in cognitive tasks performance with BMI as a covariate is shown in Table 3. The three groups showed significant differences in the performance of TMT-B time and TMT-B errors, but no significant differences in the performance of other tests. The post-multiple comparisons results are shown in Figure 1. For the result of TMT-B time, the completion time of non-users was 187.28 ± 71.61 s, which was significantly faster than 249.36 ± 94.53 s of short-term users (*p* = 0.002) and 227.48 ± 113.52 s of long-term BZD users (*p* = 0.041). As for TMT-B errors, the number of errors of short-term BZD users was 1.85 ± 1.86, which was significant higher compared to both the non-users (number of errors = 0.64 ± 0.96, *p* < 0.001) and the long-term users (number of errors = 0.87 ± 1.24, *p* = 0.001).

**Table 3:** Results of Analysis of Covariance (ANCOVA) of differences in the cognitive tasks results with BMI as covariate.

**Test**	**Variable**	**Non-users**	**Short-term BZD users**	**Long-term BZD users**	***F*-value**	***P-value***
MMSE	Total scores	27.48 ± 2.64	25.90 ± 4.79	27.30 ± 2.60	1.71	0.186
AVLT	AVLT N1	3.98 ± 1.53	3.43 ± 1.80	3.51 ± 1.60	1.18	0.311
	AVLT N2	5.30 ± 1.60	5.11 ± 2.08	4.91 ± 1.91	0.49	0.612
	AVLT N3	6.40 ± 1.88	6.09 ± 2.50	5.77 ± 1.90	1.06	0.349
	AVLT N4	4.94 ± 2.65	4.00 ± 2.55	4.11 ± 2.43	1.53	0.221
	AVLT N5	19.80 ± 5.33	18.96 ± 5.57	19.64 ± 3.91	0.17	0.841
TMT	TMT A time	73.49 ± 51.85	91.08 ± 52.17	83.15 ± 57.77	0.62	0.542
	TMT A errors	0.62 ± 2.27	0.61 ± 0.99	0.64 ± 1.17	0.12	0.886
	TMT B time	187.28 ± 71.61	249.46 ± 94.53	227.48 ± 113.52	5.23	0.006^*^
	TMT B errors	0.64 ± 0.96	1.85 ± 1.86	0.87 ± 1.24	9.68	0.000^*^
COWAT	COWAT 15s	8.67 ± 2.76	9.48 ± 3.34	8.81 ± 2.43	1.24	0.293
	COWAT 60s	19.56 ± 4.98	19.23 ± 4.08	18.95 ± 5.31	0.18	0.836
DST	DST forwards	12.41 ± 2.23	11.39 ± 1.88	11.82 ± 2.37	2.88	0.059
	DST backwards	5.34 ± 1.15	4.89 ± 1.26	4.92 ± 1.42	1.80	0.169
BVMT-R	BVMT-R N1	5.73 ± 1.82	5.22 ± 1.57	5.15 ± 2.24	1.31	0.273
	BVMT-R N2	8.09 ± 2.48	7.83 ± 2.74	6.98 ± 2.55	1.88	0.158
	BVMT-R N3	9.21 ± 2.74	9.09 ± 2.86	7.95 ± 2.82	2.19	0.118

* *Result statistically significant; MMSE, Mini-Mental State Examination; AVLT, the Rey Auditory Verbal Learning Test (AVLT N1-N4 = the number of words the participant can repeat correctly each time, AVLT N5 = the number of words the participant can repeat correctly 5 min after N4); TMT, the Trail Making Test; COWAT, the Controlled Oral Word Association Test (COWAT 15 s/60 s = the number of words the participant can speak in 15 s/60 s); DST, the Digit Span Test; BVMT-R, the Brief Visuospatial Memory Test-Revised (BVMT N1-N3 = the scores the participant can gain each time)*.

Consistent with our prediction, multiple linear regression analyses showed no significant decline in the cognitive tests of MMSE, AVLT, TMT, COWAT, and DST with longer duration of BZD use. Interestingly, as shown in Table 4, the duration of BZD use was positively correlated to better performance in the BVMT-R N1 test (*r* = 0.235, *p* = 0.023). After adjusted for demographic variables, the BVMT-R N1 scores of BZD users were still correlated with BZD over time (*r* = 0.377, *p* = 0.012).

**Table 4:** Multiple linear regression analysis for the factors determining BVMT-R N1 scores in BZD users.

**Model**	**Beta**	***t***	***P***	***R***	***R*^**2**^**	**Adjusted *R*^**2**^**
Crude	0.235	2.306	0.023	0.235	0.055	0.045
Model 1	0.256	2.553	0.012	0.377	0.142	0.092

*Model 1: adjusted for age, sex, education, and BMI*.

## Discussion

In this study, we explored the association between BZD use and the cognitive functioning of highly educated older adults by comparing performance in cognitive tasks for long-term BZD users, short-term BZD users, and non-users. Within our study, the two BZD use groups and the control group were matched in most demographic information, including age, gender, education, smoking status, and drinking status. There was, however, a significant difference in the BMI of the three groups, with BZD user groups having the lowest BMI scores. This is consistent with participants' demographic information in a previous study on BZD use (42). With the BMI value as a covariate, the main results of this study include: (1)There were significant differences in the executive function of TMT-B test among the three groups, while there were no significant differences in the scores of MMSE reflecting general cognition, AVLT reflecting verbal memory, COWAT reflecting language fluency, DST reflecting attention, and BVMT-R reflecting visual memory; and (2) Within highly educated older adults using BZD, the duration of medication was significantly correlated with a higher BVMT-R N1 score.

Comparing to other studies on BZD use and cognitive functioning, some demographic data of our study were inconsistent. For example, some previous studies included more women in the BZD use group than the non-use group (5, 6). In our study, there was no significant difference in gender distribution between the BZD use and non-use groups. There are two possible reasons for this discrepancy. The small sample size of our study might not accurately reflect the demographic attributes of the entire patient population. Another possible explanation for the inconsistency may be related to the specific participants group in our study. It is likely that the population of highly educated adults has the same sex ratio in the subgroups of BZD short-term users, BZD long-term users, and non-users. Therefore, the restriction to high education in sample selection reduced the difference of demographic information between the BZD condition groups.

### BZD Use and the Altered Cognition Domains in Highly Educated Older Adults

In the ANCOVA result for differences in cognitive tasks, the three groups only showed significant differences in the completion time and number of errors in the TMT-B test. Previous research has shown that although TMT-B time and TMT-B errors can both reflect participants' executive function, TMT-B time has a stronger correlation with visual movement, while the performance of TMT-B errors has a stronger correlation with working memory (35, 36). In the regression analysis, there was a significant correlation between BZD using time and BVMT-R N1 score reflecting immediate visual memory. These results suggest that the effects of BZD use on the cognitive function of highly educated old adults may be mainly manifested in the immediate visual memory function and executive function including visual motor and working memory.

Although there has been no previous research on the specific cognitive domains affected by BZD use in highly educated older adults, some research on general aged BZD users has reported that the cognitive effects of BZD exist in a variety of cognitive domains including executive function and visual memory (14, 43–48). A 7-year cohort study in France by Mura et al. (44) suggested that there was a significantly associated between BZD use and lower performance of immediate visual memory and executive function, but no significant association between BZD use and lower global cognitive function in older adults. In their study, 29.6% non-users and 33.8% BZD users had 9–12 years of education, and 40.9% non-users and 32.1% chronic users had at least 12 years or more of education (44). In addition, some studies' results were not entirely consistent with our findings. Ros-Cucurull et al. found that auditory memory function and visual-spatial ability of BZD users were also significantly impaired compared to non-users with 75% of participants' education level fewer than 10 years (45). In a study with participants with an average education level of <9 years, Helmes and Ostbye found that auditory memory functioning in BZD users was significantly impaired compared with that of non-users (47). These inconsistencies may be related to the high educational level of the participants in our study (14.88 ± 3.19 years). The inconsistency across studies of cognitive performance tests in highly educated older adults may be because of the protective effects of higher education on certain cognitive domains. Batterham's study showed that higher education affected cognition decline differently across the domains. They explored the protective effects of education in three cognition domains, including global cognition, processing speed, and memory, and found that higher education was associated with worse global cognition but not memory and processing speed (49). Therefore, the nature and mechanism of higher education's protective effects are still unclear.

### BZD Using Time and the Cognition Decline in Highly Educated Older Adults

In this study, compared to non-users and long-term BZD users, short-term BZD users showed a significant increase in TMT-B errors, reflecting worse working memory function. At the same time, there was no significant difference between the non-users and the long-term BZD users. It was also found that the numbers of TMT-B errors in long-term BZD users were significantly fewer than that of the short-term BZD users, and there was no significant difference between the long-term BZD users and the non-users. In addition, although there was no significant difference in BVMT-R scores among the three groups, there was a significant correlation between the number of BVMT-R N1 scores and the BZD using time among all BZD users. These results suggest that the negative cognitive effects of BZD (at least in the domain of immediate visual memory and executive control) in the highly educated older adults did not increase with longer duration of BZD use. Previous studies have shown that long-term BZD use might result in physical dependence (2), and patients with BZD dependence could significantly impair cognitive functioning (25). Exploring the protective factors on the progress of cognition decline in dependent patients may help prevent severe cognitive impairment and improve an individual's prognosis. The results of our study may provide a new perspective to explore the protective effects of high education level on BZD dependence. However, since this study is a cross-sectional study and there is no follow-up data, it should be interpreted with caution.

Past research on general aged BZD users is consistent with our findings (14, 44, 46, 50). A prospective study of 3,434 BZD users over 10 years by Gray et al. showed that the risk of dementia in the older adults increased and then decreased with increased BZD use (46). The results of Zhang et al. 7-year cohort study of 5,423 BZD users with an average education level of 15 years found no faster decline of general cognition reflected by MMSE with long-term BZD use (14). A double-blind randomized controlled study conducted by Voshaar et al. (51) observed the long-term BZD users' resilience of the negative cognition effect by BZD. After taking 10 mg or 30 mg diazepam 2.5 h, long-term users can almost completely tolerate acute effects on cognition, but the control group still showed cognitive damage (50). A 7-year cohort study of 5,195 BZD users by Mura et al. showed no longitudinal association between long-term BZD use and accelerated cognitive decline (44). The phenomenon that the cognitive impairment of BZD users did not increase with the duration of medication may not be due to their high education level as previous research has shown (52, 53). One possible explanation was that after a period of the therapeutic use of BZD, the elevated mental health of the BZD users can promote the cognition (54). However, some research showed that aged BZD users suffered more cognitive impairment as their time on the drug increased (51, 55–57). These inconsistencies may be due to researchers not considering that BZD users had higher levels of anxiety than the control group, which results in a reduction in cognitive functioning (26). This explanation is supported by the research of Lucki et al., which reported that the cognitive performance of long-term BZD users did not differ significantly from that of non-users after matching for age, gender, education, and anxiety levels (58).

### Strengths and Limitations

This study aims to assist with the clinical decision making of prescribing BZD to older adults. The therapeutic effects of BZDs are widely supported, but a major concern with BZD treatment is its potential negative effects on patients' cognitive functions, especially in older patients. With the increase in the accessibility of higher education, as well as the prevalence of BZD prescription, it is expected that there will be an increasing population of highly educated older adults using BZD. This research directly responds to these growing trends, attempting to address an expected clinical question: Are older patients less at risk for the negative cognitive effects BZD treatment's if the patients are highly educated? This forward-seeing perspective of this study provides unique strength and clinical meaning. This study focused on the population of highly educated old adults when investigating the cognitive effect of BZD and controlled for several demographic and clinical variates. Other than BMI, all other demographic information of the groups well-matched.

However, there are also some limitations. First, although BZD users were divided into long-term users and short-term users to explore the relationship between the BZD use duration and BZD's cognition effects, as a cross-sectional investigation, this study cannot directly reflect the causal relationship between BZD use and how did the BZD's cognition effects change with using time. Second, due to the outbreak of COVID-19 in China in December 2019, the study had to be discontinued, leaving the sample size of the study unsatisfactory. Third, we did not consider the role of sleep in the cognition effect of BZD. Past research has shown that sleep could influence cognitive performance in older adults (59) While many older adults use BZD as a treatment for insomnia (5), this study did not assess the participants' sleep quality. Therefore, the differences in cognitive outcomes might be due to the differences in sleep quality among the three groups. Further research is needed to exclude the influence of sleep. Additionally, some other factors related to older adults' cognition, such as occupation and daily activity, might also have influenced the results of the study.

This current study inspires multiple options for future research. The next step of this project could be to design an experimental study that assigns BZD non-users into BZD use and other anxiolytics groups to further reduce the curative effect of the drug on the cognition outcomes. A longitudinal study can also be informative in providing insight into the causality and long-term effects of BZD use. To eliminate the effects of depression and anxiety on cognitive performance, we excluded patients with those conditions. However, because BZD is widely prescribed to manage mood disorders, it is clinically relevant to include those populations in future studies. A preliminary proposal includes a within-subject design examining the cognitive performance of mood disorder patients before and after the onset of BZD treatment. This current study investigated the relationship between BZD use and cognitive performance. Other side-effects of BZD use such as addictive effects should also be evaluated in the context of highly educated adults.

## Conclusion

In conclusion, we investigated the cognitive effects of BZD use on highly educated older adults and obtained valuable clinical findings. The results of this study found that in the population of highly educated older adults, short-term BZD use is significantly associated worse executive function (TMT), but functioning in general cognition (MMSE) and other cognitive domains such as verbal memory (AVLT), language fluency (COWAT), attention (DST), and visual memory (BVMT-R) was not associated with BZD use. Moreover, the cognition effect of BZD in highly educated old did not increase with the duration of BZD use. The result of this study can contribute to the discourse of the security of BZD use among older adults and provides guidance on clinical medicine in the future.

## Data Availability Statement

The raw data supporting the conclusions of this article will be made available by the authors, without undue reservation.

## Ethics Statement

The studies involving human participants were reviewed and approved by the ethics committee in the First Affiliated Hospital of China Medical University. The patients/participants provided their written informed consent to participate in this study.

## Author Contributions

LL and YT: conceptualization. LL and MT: data curation and analysis. RZ and YT: project administration. LL, YZ, LJ, and PJ: supervision, writing—review, and editing. LL and PJ: writing—original draft. All authors contributed to the article and approved the submitted version.

## Conflict of Interest

The authors declare that the research was conducted in the absence of any commercial or financial relationships that could be construed as a potential conflict of interest.
