# Supplementary material for: Is the Long-Term Use of Benzodiazepines Associated With Worse Cognition Performance in Highly Educated Older Adults?
Source: Front Psychiatry. 2020 Oct 26;11:595623. doi: 10.3389/fpsyt.2020.595623 (PMC7649772; doi:10.3389/fpsyt.2020.595623)
Supplement: Supplementary file 1 [file Data_Sheet_1.docx]

Supplementary Material

#
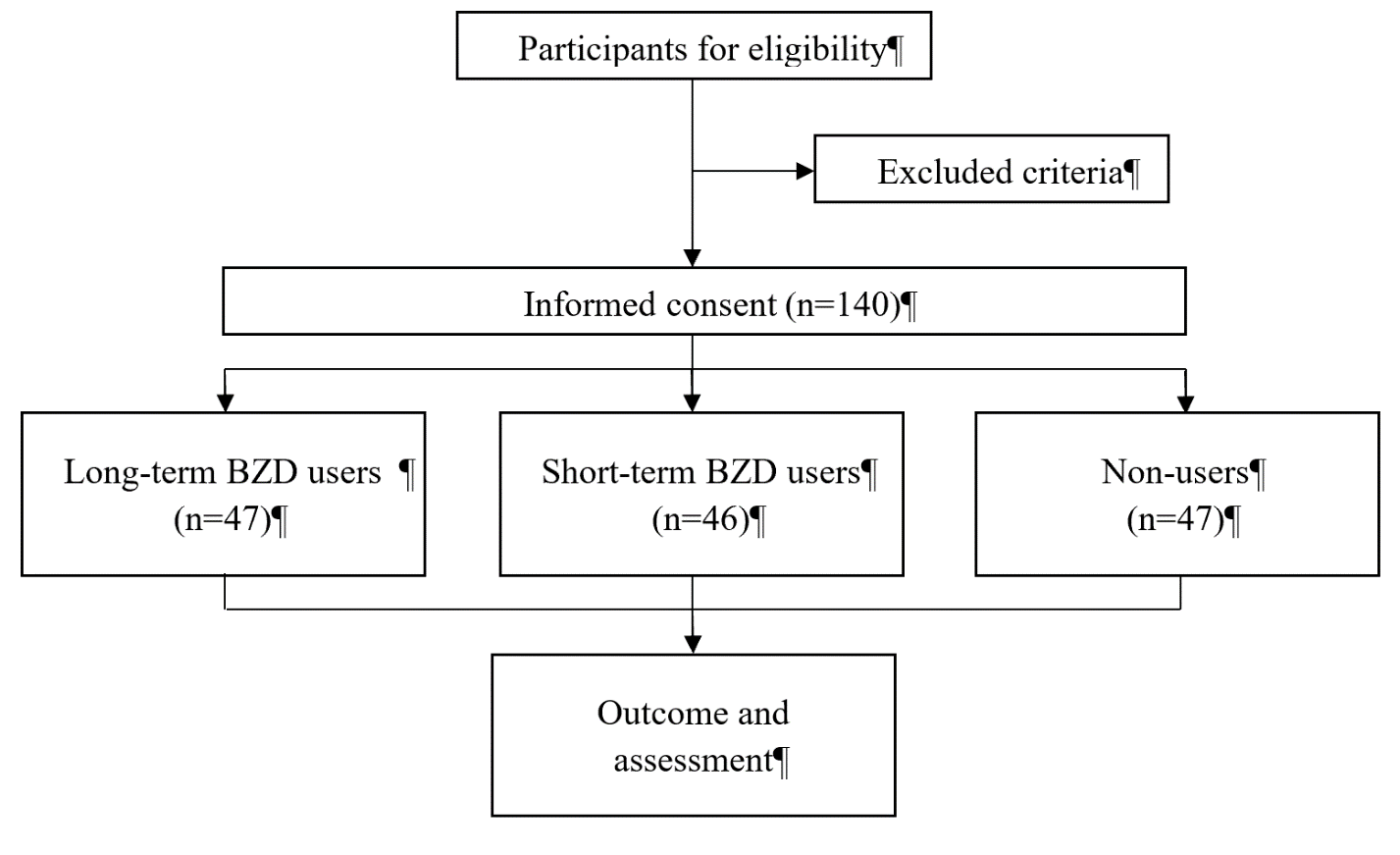
Supplementary Figures

**Supplementary Figure 1.** Experimental flow chart.
